# The Lectin Pathway of Complement in Myocardial Ischemia/Reperfusion Injury—Review of Its Significance and the Potential Impact of Therapeutic Interference by C1 Esterase Inhibitor

**DOI:** 10.3389/fimmu.2018.01151

**Published:** 2018-05-25

**Authors:** Anneza Panagiotou, Marten Trendelenburg, Michael Osthoff

**Affiliations:** ^1^Division of Internal Medicine, University Hospital Basel, Basel, Switzerland; ^2^Department of Biomedicine, University Hospital Basel, University of Basel, Basel, Switzerland

**Keywords:** C1 esterase inhibitor, complement system, complement inhibition, ischemia/reperfusion injury, mannose-binding lectin, myocardial infarction, inflammation

## Abstract

Acute myocardial infarction (AMI) remains a leading cause of morbidity and mortality in modern medicine. Early reperfusion accomplished by primary percutaneous coronary intervention is pivotal for reducing myocardial damage in ST elevation AMI. However, restoration of coronary blood flow may paradoxically trigger cardiomyocyte death secondary to a reperfusion-induced inflammatory process, which may account for a significant proportion of the final infarct size. Unfortunately, recent human trials targeting myocardial ischemia/reperfusion (I/R) injury have yielded disappointing results. In experimental models of myocardial I/R injury, the complement system, and in particular the lectin pathway, have been identified as major contributors. In line with this, C1 esterase inhibitor (C1INH), the natural inhibitor of the lectin pathway, was shown to significantly ameliorate myocardial I/R injury. However, the hypothesis of a considerable augmentation of myocardial I/R injury by activation of the lectin pathway has not yet been confirmed in humans, which questions the efficacy of a therapeutic strategy solely aimed at the inhibition of the lectin pathway after human AMI. Thus, as C1INH is a multiple-action inhibitor targeting several pathways and mediators simultaneously in addition to the lectin pathway, such as the contact and coagulation system and tissue leukocyte infiltration, this may be considered as being advantageous over exclusive inhibition of the lectin pathway. In this review, we summarize current concepts and evidence addressing the role of the lectin pathway as a potent mediator/modulator of myocardial I/R injury in animal models and in patients. In addition, we focus on the evidence and the potential advantages of using the natural inhibitor of the lectin pathway, C1INH, as a future therapeutic approach in AMI given its ability to interfere with several plasmatic cascades. Ameliorating myocardial I/R injury by targeting the complement system and other plasmatic cascades remains a valid option for future therapeutic interventions.

## Introduction

Ischemic heart disease is still a major cause of morbidity and mortality worldwide. In the United States, more than 700,000 episodes of acute myocardial infarctions (AMIs) are diagnosed annually (1). AMI is the consequence of rupture or erosion of a vulnerable atherosclerotic plaque in the coronary arteries subsequently leading to total or partial occlusion and tissue ischemia. In patients with total occlusion, emergency reperfusion of ischemic myocardial tissue is the cornerstone of therapy to salvage ischemic tissue from permanent damage. However, abrupt restoration of coronary blood flow with reperfusion of ischemic myocardium may itself trigger additional injury, which is known as ischemia/reperfusion (I/R) injury and may lead to the death of previously viable cardiac tissue (2). Estimates from previous experimental studies suggest that I/R injury may account for a significant (up to 50%) proportion of the final infarct size (3). Several mechanisms and mediators of I/R injury have been previously identified including oxidative stress, inflammation, and endogenous salvage kinase pathways (4). Despite promising results in experimental and early phase human studies (5, 6), interventional strategies targeting cardiac I/R injury have not been successful including the phase 3 trials of cyclosporine or pexelizumab before percutaneous coronary intervention in patients with AMI (7, 8).

Regarding inflammation as one mediator of I/R injury, experimental and clinical studies have shown that reperfusion after transient ischemia results in activation of endothelial cells, the contact and the complement system and attraction of neutrophils to the site of infarction (9, 10). The complement system is a major component of innate immunity, which is involved in both recognition and response to pathogens (11). It is further implicated in an increasing number of homeostatic and disease processes such as the immune complex catabolism, the clearance of dead and dying cells and the modulation of adaptive immune responses (12). Three pathways can activate the complement cascade: the classical, the alternative, and the lectin pathway. After initiation, these three pathways converge at the level of cleavage and activation of complement component C3, which subsequently leads to the generation of the anaphylatoxins (C3a, C5a) and the membrane attack complex (MAC; C5b-9). This review focuses on the role of the lectin pathway in myocardial I/R injury and the potential benefit of therapeutic application of its natural inhibitor, C1 esterase inhibitor (C1INH). In particular, we will focus on the potential advantages of C1INH as a promising candidate for future trials of salvage strategies of hypoxic myocardial tissue after AMI.

## The Lectin Pathway of Complement

The lectin pathway can be activated by the pattern-recognition receptors (PRR), mannose-binding lectin (MBL), ficolin-1, ficolin-2, ficolin-3, collectin 10 (CL-10), and collectin 11 (CL-11 or CL-K1). These glycoproteins bind to carbohydrate patterns, acetyl groups, or immunoglobulin M exposed on microorganisms or dying host cells (13, 14). In plasma, lectin pathway PRR complex together with MBL-associated serine proteases (MASPs)-1, -2, -3, and two non-protease peptides, sMAP (Map19) and MBL/ficolin-associated protein-1 (MAP-1 or Map44). Whereas MAP-1 was shown to be a natural inhibitor of the lectin pathway (15), the exact function of sMAP is still unknown. After binding of the PRR to their target structures, MASP-1 is activated which in turn activates MASP-2, which is required for generating the C3 convertase (C4b2a) (16). The C3 convertase cleaves C3 into the opsonin C3b and the anaphylatoxin C3a, initiating the formation of an additional anaphylatoxin (C5a) and the MAC (C5b-9). Whereas the function of MASP-2 is strictly limited to the activation of the lectin pathway, several proteolytic functions have been observed for MASP-1, such as cleavage of fibrinogen, factor XIII, prothrombin and thrombin-activated fibrinolysis inhibitor (coagulation cascade), cleavage of kininogen (kallikrein-kinin cascade), and activation of protease-activated receptors on endothelial cells (neutrophil attraction) (17). Although the proteolytic activity of MASP-1 toward these proteins is much lower compared with the primary cleaving or activating enzymes of these proteins or compared with the primary lectin and classical pathway target proteins of MASP-1, it may still be of relevance *in vivo*. For example, a prolonged bleeding time and decreased arterial thrombogenesis was observed in a MASP-1 knock-out rodent model (18, 19). In the context of AMI, activation of the lectin pathway of complement after AMI may impact on I/R injury not only through activation of the complement cascade but also *via* promotion of clot formation (coagulation and fibrinolytic system) and of inflammation (kallikrein-kinin cascade, activation of endothelial cells, and attraction of neutrophils). Hence, inhibition of the lectin pathway, particularly at the level of MASP-1/-2 seems to be advantageous over downstream inhibition of the complement.

## C1 Esterase Inhibitor

**Figure 1:**
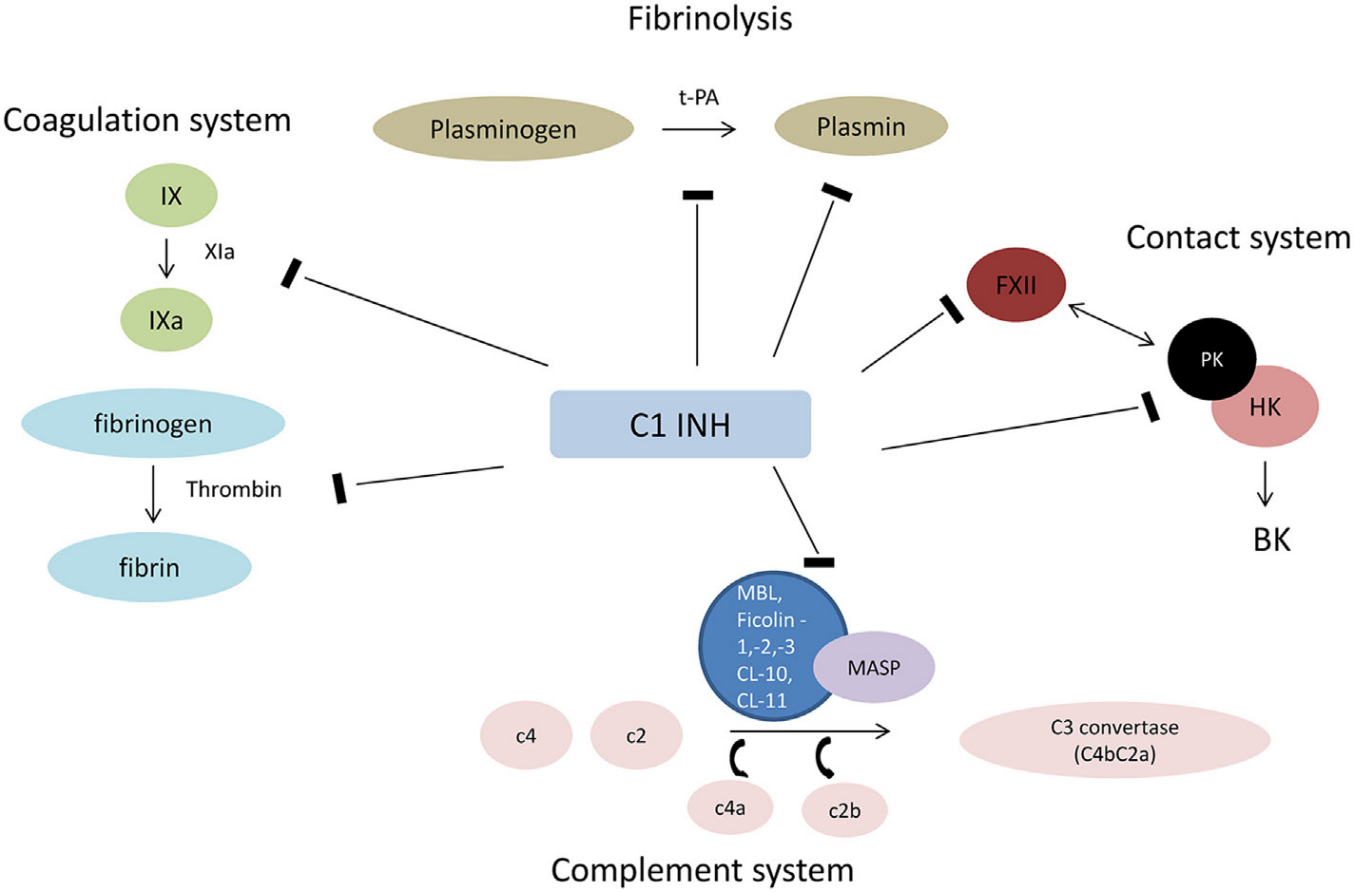
Interaction of C1INH with plasmatic cascades. Abbreviations: HK, high molecular weight kininogen; FXII, factor XII (Hageman factor); PK, prokallikrein; BK, bradykinin; t-PA, tissue plasminogen activator; MAC, membrane attack complex.

The most important natural inhibitor of the lectin pathway of complement is C1INH. C1INH, a member of the serpin superfamily of serine protease inhibitors, is an acute-phase protein that has manifold targets and biological functions. Although the primary function of serpins involves the inhibition of proteases, they are also implicated in additional biological interactions, such as the inhibition of leukocyte rolling and interactions with endothelial cells and microorganisms (20). For example, treatment with C1INH was shown to limit the activation of endothelial cells and their subsequent transition into a procoagulatory and antifibrinolytic state after I/R injury (21). Proteases that are inactivated by C1INH include C1r, C1s (classical pathway of complement), MASP-1 and MASP-2 (lectin pathway), factor XII and plasma kallikrein (contact system), factor XI and thrombin (coagulation system), and plasmin and tissue plasminogen activator (fibrinolytic system) (22, 23) (Figure 1). Binding of C1INH to any of its target proteases leads to tight complexes which are subsequently cleared from the circulation and can be summarized as suicide inhibition (9). Decreased plasmatic antigenic levels of C1INH result in uncontrolled production of vasoactive peptides, which leads to the characteristic episodes of local soft tissue swelling observed in hereditary angioedema (HAE) (24).

Regarding the complement system, MASP-1 and -2 seem to be the major target of C1INH with less effective inhibition of the classical pathway (25). Interestingly, the lectin pathway and in particular MASP-1 have been recently implicated in the pathophysiology of HAE, which underscores the central role of C1INH in controlling the activation of the lectin pathway. C1INH deficiency seems to cause uncontrolled activation of MASP-1, which may aggravate HAE (26).

Currently, three C1INH preparations are approved for the treatment and/or prevention of HAE, two plasma-derived, pasteurized, and nanofiltered (pdC1-INH, Berinert^®^ and Cinryze^®^), and one recombinant product [rhC1-INH, Conestat alfa (Ruconest^®^), derived from the breast milk of transgenic rabbits]. Conestat alfa shares an identical protein structure with plasma-derived C1INH, but has a different glycosylation pattern of the amino-terminal domain of the protein (containing abundant oligomannose residues), which is responsible for a markedly shorter half-life compared with plasma-derived C1INH (3 vs. 30 h) (27). In fact, the unique glycosylation pattern introduced by the production of Conestat alfa in the mammary gland of transgenic rabbits may have additional, yet undiscovered consequences. For example, artificial variation of the glycosylation pattern of pdC1INH was previously shown to selectively impact on its target proteases with little impact on C1s inhibition in contrast to its interaction with kallikrein (28). Moreover, an important regulatory function of the amino-terminal domain of C1INH has been identified preventing inhibition of MASP-1-mediated alternative pathway activation (29), which may be influenced by the glycosylation pattern of C1INH. Although comparable inhibition for most target proteases was demonstrated (including C1s, factor XIa, XIIa, and Kallikrein) (30), Conestat alfa seems to target MBL and activation of the lectin pathway more effectively compared with plasma-derived preparations (31). This may be related to the fact that oligomannose-type glycans on average account for 15% of the total amount of glycans of Conestat alfa (compared with less than 1% in pdC1INH) (32), which may expedite the targeting and subsequent inhibition of MBL-MASP-1/MASP-2 complex by Conestat alfa.

Despite the rather broad interference with several cascades and targets, major adverse events or unique toxicities have not been demonstrated in previous studies, with the exception of a potential risk of allergic reactions in patients with rabbit dander allergy receiving Conestat alfa. Previous concerns of an increased thrombotic risk of pdC1INH (33) have not been confirmed in recent trials and registry data of both, pdC1INH and rhC1INH (34, 35). Common side effects described in trials include headache, nausea, and diarrhea. Currently, C1INH is evaluated in interventional clinical trials in the context of transplantation, acute antibody-mediated rejection after transplantation, and contrast-induced nephropathy.

## The Lectin Pathway in Experimental Myocardial I/R Injury

Many animals and a limited number of human studies support the concept, that activation of the complement system and in particular the lectin pathway contributes to tissue injury observed after reperfusion of ischemic myocardial tissue (Table 1). Collard et al. were the first to report lectin pathway-dependent local activation of the complement system after myocardial I/R injury (36). Co-localized deposition of MBL and C3 in rat hearts was detected following myocardial I/R but not after sham surgery or myocardial ischemia only. Shortly thereafter, the same group demonstrated in a rat model of myocardial I/R injury that selective inhibition of MBL-A decreased infarct size and limited tissue injury and C3 deposition (37). In addition, infiltration of reperfused myocardial tissue by neutrophils was attenuated. Walsh et al. were the first to demonstrate the crucial involvement of the lectin pathway when compared with the classical and alternative pathway in myocardial I/R injury (38), an important finding, which contradicted earlier studies that had implicated the classical pathway as the most important mediator of tissue injury after I/R (39). According to Walsh et al., myocardial I/R injury was strongly attenuated in MBL knock-out mice, whereas mice with an intact lectin pathway but lacking C1q, the PRR of the classical pathway, or factor D of the alternative pathway were not protected. Reconstitution with recombinant MBL abrogated the protective effect. These results were replicated in a mouse model of diabetes with two weeks of hyperglycemia followed by myocardial I/R injury (40).

**Table 1:** Experimental studies investigating the role of the lectin pathway in murine myocardial I/R.

Reference	Intervention/setting	I/R model	Species	Effect
Collard et al. (36)	n/a	30 min LAD occlusion, 30 min reperfusion 60 min LAD occlusion	Rat	I/R: strong C3 and MBL staining throughout ischemic area Ischemia only: minimal C3 or MBL deposition
Jordan et al. (37)	Anti-MBL-A mAbs (P7E4, 14C3.74)	30 min LAD occlusion, 4 h reperfusion Pretreatment with mAbs (5 min before ischemia)	Rat	P7E4: ↓ local C3 deposition, infarct size, accumulation of PMN and expression of pro-inflammatory genes 14C3.74: no effect
Walsh et al. (38)	MBL−/− C1q−/− C2/fB−/− fD−/− C1q/fD−/− Anti-C5 mAb	30 min LAD occlusion, 3 h reperfusion pretreatment with mAb (5 min before ischemia) Reconstitution with C2 or rhMBL	Mouse	MBL−/−, C2−/−, anti-C5 mAb: ↓ infarct size, effect lost after reconstitution with rhMBL or C2 C1q−/−, C1q/fD−/−, fD−/−: no effect; co-localization of MBL and C3 in ischemic area
Busche et al. (40)	Streptozotocin-induced diabetic MBL−/−	15 or 30 min LAD occlusion, 4 h reperfusion	Mouse	↓ hyperglycemia induced myocardial remodeling and loss of cardiac progenitor cells ↓ cardiac tissue injury and local PMN infiltration after I/R injury
Busche et al. (41)	sIgM/MBL−/−	30 min LAD occlusion, 4 h reperfusion reconstitution with MBL null, sIgM null, or sIgM/MBL null plasma	Mouse	↓ cardiac tissue injury, loss of ventricular function, local C3 deposition and PMN infiltration; effect lost after reconstitution with MBL null, sIgM null or sIgM/MBL null plasma
Schwaeble et al. (42)	MASP-2−/− C4−/−	30 min LAD occlusion, 2 h reperfusion	Mouse	MASP-2−/−: ↓ infarct size and cardiac tissue injury C4−/−: no effect
Pavlov et al. (15)	MBL−/− MAP-1	45 min LAD occlusion, 4 h reperfusion pretreatment with rhMBL and/or MAP-1	Mouse	MAP1: ↓ loss of ventricular function, infarct size and local C3 deposition; prevention of occlusive arterial thrombogenesis MBL−/−: ↓ loss of ventricular function and infarct size
Pavlov et al. (44)	hMBL2 expressing mouse Anti-MBL2 mAb	45 min LAD occlusion, 4 h reperfusion treatment with mAb at or 15 and 30 min after reperfusion	Mouse	↓ loss of ventricular function, infarct size (time dependent protection) and myocardial fibrin deposition; prevention of occlusive arterial thrombogenesis
Clark et al. (43)	Anti-MASP-2 mAb	30 min LAD occlusion, 2 h reperfusion pretreatment with mAbs (12–18 h before ischemia)	Mouse	↓ infarct size

*fB, factor B; fD, factor D; I/R, ischemia and reperfusion; LAD, left anterior descending artery; mAbs, monoclonal antibodies; MBL, mannose-binding lectin; rhMBL, recombinant human MBL; PMN, polymorphonuclear leukocytes; sIgM, secreted immunoglobin*.

As a previous study had suggested a sequence of binding of IgM to stressed endothelial cells followed by complement activation of the lectin pathway, the same group studied myocardial I/R injury in reconstitution experiments using mice lacking natural IgM and MBL (41). Interestingly, both, MBL and IgM were found to be required for increased myocardial C3 deposition, neutrophil infiltration, and loss of left ventricular function after reperfusion of ischemic myocardial tissue. Hence, binding of natural IgM to neoepitopes in ischemic tissue was suggested as a prerequisite for subsequent MBL-mediated complement activation and tissue injury.

Schwaeble et al. investigated myocardial tissue injury in MASP-2 and C4-deficient mouse models, i.e., in the absence of any residual lectin (MASP-2) and presumably of any classical/lectin pathway activity (C4), and further downstream after binding of MBL or other lectin pathway PRR to stressed and injured cardiomyocytes (42). Lack of MASP-2 but not of C4 led to significantly less I/R-induced myocardial damage, which suggests that MASP-2-mediated activation of the lectin pathway is a major requirement for the inflammatory myocardial tissue damage after I/R. Activation of the two other complement pathways does not seem to be sufficient in the absence of any lectin pathway activity. Importantly, the authors identified a MASP-2-dependent, C4-independent route of complement activation, which likely also involves MASP-1 (29). This finding highlights the importance of inhibiting the activation of the complement system as far upstream as possible. In line, treatment of mice with an anti-MASP-2 monoclonal antibody (mAb) administered 12–18 h before coronary artery occlusion and reperfusion led to significantly smaller infarct sizes (15 vs. 26% in isotype control treated animals) (43). Similarly, treatment with MAP-1, an endogenous lectin pathway inhibitor, which displaces MASP-2 from the MASP-MBL/ficolin-3 complex and inhibits MBL- and ficolin-3-dependent complement activation *in vitro*, was associated with a decreased infarct size and C3 deposition in both, wild-type (WT) and MBL-supplemented MBL knock-out mice (15).

Finally, Pavlov et al. confirmed the concept of targeting MBL to attenuate myocardial I/R injury in a humanized mouse model (44). These humanized mice produce functional human MBL while lacking murine MBL-A and -B. Inhibition of human MBL by a monoclonal anti-human MBL antibody preserved cardiac function after myocardial I/R, attenuated myocardial fibrin deposition, and prevented ferric chloride-induced arterial thrombogenesis.

Summarizing the evidence from experimental models, the lectin pathway was shown to be crucially involved in mediating myocardial I/R injury. Conceptually, myocardial I/R injury seems to be initiated by binding of natural IgM to neoepitopes on stressed or dying cardiomyocytes with subsequent binding of MBL to IgM and activation of MASP-1/-2 during reperfusion. The latter is a major requirement for subsequent complement-mediated inflammation including formation of the membrane-attack complex, induction of apoptosis, and attraction of neutrophils. The significance of other lectin pathway PRR in acute myocardial I/R injury remains to be determined.

## The Lectin Pathway in Human Myocardial I/R Injury

Inter-individual serum concentrations of lectin pathway PRR and proteases show a considerable degree of variability in humans (45). In particular, the distribution of MBL plasma levels is huge and ranges from undetectable to approximately 10 µg/ml secondary to well-characterized exon and promoter polymorphisms in the MBL2 gene (46). Very low (<0.1 μg/ml, resembling a knock-out setting in murine models) and low (<0.5 µg/ml) MBL levels are found in approximately 10 and 30% of the population worldwide, respectively. Several studies have investigated associations of lectin pathway protein levels, in particular MBL, with outcome after AMI to confirm the evidence from murine studies (Table 2).

**Table 2:** Analysis of lectin pathway proteins in patients with AMI.

Reference	Study type	Condition	Included patients	Analyzed complement proteins	Relevant findings
Haahr-Pedersen et al. (47)	Observational	Acute STEMI	74	Plasma MBL and sC5b-9	↑ risk of cardiac dysfunction (LVEF < 35%) in patients with MBL ≥ 0.8 µg/ml and sC5b-9 ≤ 160 µg/ml
Trendelenburg et al. (48)	*Post hoc* analysis of RCT	Acute STEMI	890	Serum MBL	↓ 30-day mortality in patients with MBL deficiency (≤0.1 μg/ml); no association with cardiac tissue injury
Schoos et al. (50)	Observational	Acute STEMI	55	Plasma MBL, ficolin-2 and -3, MAP-1	↑ left ventricular dilatation/remodeling in patients with high ficolin-2 and combined ficolin-2/MBL or ficolin-2/MAP-1 baseline levels; no association with infarct size
Zhang et al. (51)	Observational	AMI (STEMI and NSTEMI)	AMI: 29 Healthy controls: 50 Stable CAD: 27	Plasma MASP-2 levels	↓ MASP-2 levels in patients with AMI compared with healthy individuals or stable CAD patients
Frauenknecht et al. (52)	Observational	AMI	AMI: 49 Healthy controls: 50 Stable CAD: 104	Plasma MASP-1, -2, -3, and MAP-1	↓ MASP-2 and ↑ MASP-1 levels in patients with AMI compared with healthy individuals or stable CAD patients; no difference in MASP-3 and MAP-1 levels
Holt et al. (53)	*Post hoc* analysis of RCT	Acute STEMI	STEMI: 192 Healthy controls: 140	Plasma MAP-1, MASP-1, MASP-3	↑ MASP-1,-3 and MAP-1 levels in STEMI patients compared with healthy individuals; no association with infarct size

*CAD, coronary artery disease; LVEF, left ventricular ejection fraction; MBL, mannose-binding lectin; NSTEMI, non ST-elevation myocardial infarction; RCT, randomized controlled trial; STEMI, ST elevation myocardial infarction; AMI, acute myocardial infarction*.

In a pilot study of 74 patients with ST elevation myocardial infarction (STEMI) and successful reperfusion, sufficient plasma MBL levels (defined as >0.8 µg/ml) were independently associated with a significant cardiac dysfunction [defined as left ventricular ejection fraction (LVEF) <35%] (47). In line with the concept of downstream complement activation following binding of MBL to stressed endothelial cells and/or cardiomyocytes and subsequent consumption of complement components, plasmatic sC5b-9 levels were significantly lower in patients with cardiac dysfunction. In the largest analysis to date, MBL levels were investigated in 890 patients with STEMI receiving placebo in the setting of a randomized controlled trial of the C5 inhibitor pexelizumab (48). In this study, MBL deficiency was defined as a level ≤0.1 µg/ml. Interestingly, 30-day mortality was markedly lower in MBL deficient patients (0.8 vs. 5.5%), whereas creatine kinase levels, a marker of myocardial injury and infarction size, and complement activation product levels were similar in the two groups. The authors speculate that the observed reduction in mortality was mainly driven by protection from fatal arrhythmias in MBL-deficient patients, a well-appreciated consequence of reperfusion injury (49). However, these results have not yet been confirmed in a cohort of STEMI patients undergoing percutaneous coronary interventions with contemporary stents and antithrombotic therapy.

In a small cohort of 55 STEMI patients, Schoos et al. investigated the association of ficolin-2/-3, MBL, and MAP-1 levels with left ventricular remodeling and infarct size as assessed by cardiac magnetic resonance imaging after percutaneous coronary intervention and at 6-month follow-up (50). Ficolin-2 levels significantly increased from admission to day 4 in contrast to other lectin pathway proteins, which may indicate consumption of ficolin-2 during STEMI. Higher baseline ficolin-2 levels were associated with left ventricular dilatation after STEMI. A similar finding was observed for the combination of higher ficolin-2 and MBL levels or higher ficolin-2 and lower MAP-1 levels indicating that the overall activation of the lectin pathway (rather than higher or lower levels of a single lectin pathway protein or protease) may be the key parameter influencing left ventricular dilatation after STEMI. However, lectin pathway proteins were not associated with infarct size, left ventricular function, or remodeling after 6 months, similar to the lack of association between MBL and infarct size observed in the larger study of Trendelenburg et al. (48). Zhang et al. investigated the role of MASP-2 in myocardial ischemia in the setting of AMI and separately in open heart surgery (51). For the purposes of this article, we only report the results of 29 AMI patients being described in this study. MASP-2 levels determined within two days after admission were almost 50% lower compared with healthy individuals or patients with stable coronary artery disease (CAD). This may be consistent with activation of the lectin pathway during AMI with subsequent consumption of MASP-2, similar to the previously cited observation of reduced ficolin-2 levels on admission. Although follow-up samples were lacking, it seems unlikely that genetically determined lower MASP-2 levels were already present before the AMI or that a temporary change in MASP-2 levels triggered the AMI in these patients.

In another study, lower MASP-2 and higher MASP-1 levels were observed in patients with myocardial infarction compared with patients with stable CAD and healthy controls (only MASP-1) (52), whereas MAP-1 levels were similar in these groups. However, the sample size was limited (*n* = 49 AMI patients) and protein levels were not associated with the severity of cardiovascular disease. Finally, levels of MAP-1, MASP-1, and -3 were analyzed in 192 AMI patients and 140 healthy controls (53). Whereas protease levels were significantly higher in AMI patients compared with healthy controls, they did not correlate with final infarct size or LVEF at 30 days. Importantly, results were in agreement with the previous study by Holt et al. only with regards to elevated MASP-1 levels but not with regards to observed MAP-1 levels. Again, follow-up samples were not available, and hence it remains to be determined if the observed changes in lectin pathway proteins are the consequence or the cause of the initial AMI event.

In summary, results from the cited studies indicate a potential involvement of the lectin pathway of complement in human myocardial I/R injury. In particular, levels of the PRR ficolin-2 and the activating protease MASP-2 were significantly lower in AMI patients on admission, whereas the levels of the endogenous inhibitor MAP-1 were higher (in only one of two studies), which may indicate activation of the lectin pathway during AMI with consumption of MASP-2 and ficolin-2. With regards to the observed increased MASP-1 concentrations in AMI patients, it remains to be determined if this is a result of an acute-phase reaction or if higher levels of MASP-1 may itself trigger an AMI event under certain circumstances given its clot promoting activity (54).

Importantly, associations of lectin pathway proteins with myocardial dysfunction, infarct size, and outcome were mostly lacking with the exception of an association of higher MBL levels and a higher net activation of the lectin pathway with mortality and ventricular dilatation after STEMI, respectively. However, involvement of MBL as a major initiator of the lectin pathway has not been demonstrated at the tissue level after human myocardial I/R injury to date. Future studies are required to investigate in more detail if PRR or protease levels are indeed associated with outcome after AMI and are causally involved in I/R injury in humans.

In general, evidence for the involvement of the lectin pathway in humans is much weaker than in animal studies, which is related to the limited samples size in most studies, a lack of analysis of a contemporary cohort of patients, and that most individual lectin pathway proteins have only been analyzed in a single study (with the exception of MBL, MASP-1, and MAP-1) and results have not yet been confirmed in subsequent studies. Although the significant variation of MBL levels in humans permits an analysis of complement-deficient patients in analogy to the analysis of knock-out compared with WT animals, this requires large cohorts. Evolutionary, other PRR may have evolved as a consequence of MBL deficiency and may compensate and in the case of I/R injury augment tissue injury independent of MBL. Unfortunately, experimental data regarding the significance of ficolins or collectins in myocardial I/R injury are lacking.

## C1INH in Experimental Myocardial I/R Injury

The complement system was thought to play a major role in initiating an inflammatory response secondary to ischemia and reperfusion following studies 30 years ago (55). In particular, the classical pathway had initially been implicated in reperfusion damage of the heart (56, 57). To inhibit the classical pathway and subsequent complement activation at an early step, several studies examined the effects of treatment with C1INH on myocardial tissue damage, long before the central role of C1INH in the inhibition of the lectin pathway on the one hand and the pivotal role of the lectin pathway in myocardial I/R injury on the other hand was discovered (Table 3).

**Table 3:** Effect of C1INH in murine models of myocardial I/R injury.

Reference	Intervention/setting	I/R model	Species	Effect
Buerke et al. (39)	pdC1INH	90 min LAD occlusion, 4.5 h reperfusion 75 IU/kg pdC1INH 10 min before reperfusion	Feline	↓ infarct size cardiac, tissue injury and PMN infiltration
Murohara et al. (59)	pdC1INH sCR1	20 min LAD occlusion, 24 h reperfusion 75 IU/kg pdC1INH or sCR1 1 min before reperfusion	Rat	C1INH: ↓ cardiac tissue injury and PMN infiltration sCR1: non significant decrease in cardiac tissue injury and PMN infiltration
Horstick et al. (58)	pdC1INH	60 min LAD occlusion, 2 h reperfusion Intracoronary application of 20 IU/kg pdC1INH at reperfusion	Swine	↓ infarct size, tissue injury, and local C3a production
Buerke et al. (60)	pdC1INH	20 min LAD occlusion, 24 or 48 h reperfusion 10, 50, or 100 IU/kg pdC1INH 2 min before reperfusion	Rat	100 IU/kg: ↓ tissue injury, PNM accumulation, local expression of adhesion molecules 50 and 10 IU/kg: less or no effect, respectively
Horstick et al. (33)	pdC1INH	60 min LAD occlusion, 2 h reperfusion 40, 100, or 200 IU/kg pdC1INH 10 min before reperfusion	Swine	40 IU/kg: ↓ infarct size, tissue injury and local C3a and C5a production 100 IU/kg: no effect 200 IU/kg: severe coagulopathy leading to death
Buerke et al. (70)	C1s-INH-248 pdC1INH	60 min LAD occlusion, 3 h reperfusion 100 or 200 IU/kg pdC1INH or 0.1–1 mg/kg C1s-INH-248 5 min before reperfusion	Rabbit	C1s-INH-248: ↓ infarct size, tissue injury and PNM accumulation (dose dependent) C1INH: ↓ infarct size (smaller effect than C1s-INH-248)
Schreiber et al. (71)	pdC1INH	2 h LAD occlusion with cardiopulmonary bypass after 1 h, 2 h reperfusion Approximately 15 IU/kg pdC1INH into aortic root at reperfusion	Swine	No effect on ventricular function or infarct size
Fu et al. (63, 64)	pdC1INH	30 min LAD occlusion, 3–72 h reperfusion 40 IU/kg pdC1INH before ischemia	Rat	↓ myocardial apoptosis, local C3 expression, PNM accumulation and tissue injury
Lu et al. (65)	C1INH iC1INH C1INH−/− C3−/−	30 or 60 min LAD occlusion, 4 h reperfusion 400 IU/kg 5 min before reperfusion	Mouse	C1INH and iC1INH in WT mice: ↓ infarct size, tissue injury and PMN accumulation C1INH in C1INH−/− and C3−/− mice: similar effect as in WT mice

*pdC1INH, plasma-derived C1 esterase inhibitor; iC1INH, inactive C1INH; C1s-INH-248, selective C1s inhibitor; PMN, polymorphonuclear leukocytes; cardiac CK, cardiac creatine kinase; LAD, left anterior descending artery; LAD left anterior descending artery; sCR1, soluble complement receptor 1; WT, wild-type*.

In a feline model of myocardial infarction, administration of pdC1INH before reperfusion led to a 65% reduction in cardiac tissue injury, and a markedly attenuated increase of creatine kinase compared with treatment with buffered saline solution (39). In addition, neutrophil activity/accumulation in the reperfused myocardial tissue was significantly reduced, which may be related to the interaction of C1INH with activated endothelial cells or a reduction in locally generated leukocyte chemo-attractants such as C3a and C5a. The latter was subsequently confirmed in a pig model of myocardial I/R injury (58) administering pdC1INH as an intracoronary bolus before reperfusion. Local C3a production, which markedly increased after reperfusion, was significantly attenuated in the C1INH group compared with the placebo group indicating that C1INH may indeed suppress local complement activation. In a rat model of myocardial infarction, Murohara et al. sought to investigate the differential role of the classical and alternative complement pathway in myocardial I/R (59). Rats were treated with either pdC1INH or an alternative pathway inhibitor (soluble complement receptor 1) immediately before reperfusion. Interestingly, C1INH treatment was associated with a significantly attenuated creatine kinase release and neutrophil accumulation in ischemic myocardial tissue, whereas targeted inhibition of the alternative pathway was clearly inferior in this model. Finally, Buerke et al. confirmed the cardioprotective effect of different doses of plasma-derived C1INH in a rat model (60). Release of creatine kinase and local accumulation of neutrophils was attenuated in a step-wise fashion depending on the dose of C1INH with the greatest effect observed for the highest dose (100 U/kg). In addition, expression of adhesion molecules in the affected vascular endothelium was markedly reduced after administration of C1INH, which may explain the decreased local accumulation of neutrophils (61).

As a consequence of serious thromboembolic events in 13 newborns and babies who had received 500 IU/kg of pdC1INH to prevent capillary leakage after cardiopulmonary bypass operation, Horstick et al. investigated the effect of systemic administration of different doses of plasma-derived C1INH in the same pig model as had been previously used (33). In contrast to their previous model, pdC1INH was administered intravenously 10 min before reperfusion (40, 100, or 200 IU/kg) and without concurrent heparin. Experiments with 200 IU/kg were terminated after the first three pigs had developed severe coagulation disorders. Interestingly, while 40 IU/kg of C1INH reduced infarction size to a similar degree as in the previous model (>50%), there was a lack of beneficial effect with higher doses. The severe adverse events associated with the higher dose may be related to the significant inhibition of bradykinin release and of activators of the fibrinolytic system. The latter may have been prevented by co-administration of heparin, a principal anticoagulation agent in human STEMI patients (62).

In a rat model of AMI, Fu et al. observed a reduced C3 expression and apoptosis in the affected myocardial area associated with the administration of pdC1INH (63, 64). Interestingly, the amino-terminal domain of C1INH was implicated in the anti-apoptotic effect and not the protease activity.

As C1INH is a multi-action multiple-target inhibitor, it is difficult to identify the most important pathway or target protease inhibited by C1INH and responsible for the observed beneficial effect in myocardial I/R injury. While results from previous studies consistently demonstrated a clear benefit of C1INH in myocardial I/R injury, which was at least partly attributable to its inhibitory effect on complement activation, three studies have left more questions than answers with regards to the mechanism of action of C1INH and its effectiveness in cardioprotection after myocardial I/R injury. In particular, the study by Lu et al. questioned the importance of the complement system in mediating myocardial I/R injury in comparison to other mechanisms such as neutrophil influx (65). In their mouse model, a large dose of pdC1INH (400 IU/kg) or inactive C1INH (iC1INH) was administered intravenously 5 min before coronary reperfusion. iC1INH was generated by trypsin incubation, which results in the loss of its reactive center (66) and hence its ability to inhibit target proteases such as C1s. Interestingly, both C1INH and iC1INH similarly reduced myocardial damage and myocardial neutrophil influx in WT mice. In addition, C1INH treatment was effective even in C3 knock-out mice. These data are consistent with a mainly complement-independent cardioprotective mechanism of action of C1INH with the exception of its potential influence on protease-independent actions of the PRR of the lectin pathway [such as direct induction of apoptosis (67)] or its influence on lectin proteases *via* its glycosylated amino-terminal domain. The authors speculate that C1INH mainly acts *via* the inhibition of neutrophil influx across endothelial cells interacting with selectin ligands, which may be mediated by its sugar moieties rather than its proteolytic function. Two aspects warrant further comments. First, the dose of both preparations of C1INH used in the study was very high compared with previous animal models, which may be associated with additional modes of action, particularly in the case of iC1INH. Second, while the authors’ dismissed a reaction of iC1INH with C1s of the classical pathway, their work does not fully exclude a residual protease activity of MASP-1/-2 after trypsin digestion. This may be of importance, as activation of MASP-1 in particular may mediate inflammatory actions independent of downstream complement activation (68). The beneficial effect of C1INH in the C3 knock-out model may be mediated by a similar mechanism, i.e., inhibition of MASP-1 and its pro-inflammatory, complement cascade-independent function. Third, previous work has identified C1INH functions that are independent of its proteolytic activity and mediated by its glycosylated, amino-terminal nonserpin domain, such as its interaction with the endotoxin lipopolysaccharide (69) or with selectins on endothelial cells (20). Similar nonserpin actions of C1INH on lectin pathway PRR and proteases have not yet been examined or described, but may contribute to the effect observed in the study by Lu et al. (65).

Similarly, Buerke et al. investigated the effect of a C1s-specific C1INH preparation (C1s-INH-248) in a rabbit model of myocardial I/R injury (70). This novel molecule is a specific inhibitor of the classical pathway of the complement system but does not inhibit MASP-1, kallikrein, and activated factors XII and XI. C1s-INH-248 was associated with attenuated myocardial injury as demonstrated by diminished plasma creatine kinase activity and local neutrophil accumulation. In addition, C1s-INH-248 was more effective than pdC1INH administered at doses up of 200 IU/kg. The authors speculated that cardioprotective effects of C1s-INH-248 may be related to the inhibition of the classical pathway and of the interaction of neutrophils with endothelial cells, and that the lectin pathway does not play a dominant role in their model. Again, the authors do not provide evidence of a total lack of interference of this C1s-specific C1INH with functions of the lectin pathway (in particular with MASP-2).

Finally, Schreiber et al. observed a lack of effect of intracoronary application of C1INH in a pig model of acute myocardial ischemia followed by urgent coronary bypass grafting (71). In contrast to all previous studies, C1INH did not reduce infarct size or release of creatine kinase, which may be explained by several ways. First, the dose of C1INH administered was rather low compared with previous studies (approximately 11–15 IU/kg). Second, C1INH was only administered locally and 60 min after induction of CPB. CPB is well known to systemically trigger activation of the complement system (72, 73), and hence late and local administration of C1INH was probably not able to prevent systemic (and even local) complement activation. In addition, evidence of an epicardial shunt was found which again may have impacted on the limited effect of locally applied C1INH. Ideally, C1INH should be administered before institution of CPB in models of coronary bypass grafting, as this would be practicable in the human setting, too.

In summary, significant cardioprotection by C1INH was evident in all but one experimental myocardial I/R injury studies. Limitations include the use of different doses, routes of administration, and AMI models with different durations of ischemia and reperfusion. However, due to its manifold actions, the exact mechanism of C1INH being responsible for the observed attenuated injury remains to be elucidated. Apart from the inhibition of complement activation, attenuated infiltration, and accumulation of neutrophils in ischemic tissue and a reduction of endothelial cell activation may be the dominant modes of action of C1INH. Unfortunately, experimental studies examining the impact of C1INH administration on the lectin pathway of complement and its consequences in myocardial I/R models are lacking. This is of importance, as a link between the lectin pathway of complement and activation of endothelial cells and subsequent recruitment of leukocytes has been previously demonstrated (16, 17, 68), and protease-independent actions of the PRR of the lectin pathway [such as direct induction of apoptosis (67) or amplification of inflammation *via* the alternative pathway] may play a role in myocardial I/R injury. A limitation of modified C1INH preparation as used in previous studies is that the difference in its function compared with original C1INH has not been comprehensively elucidated, in particular toward its effect on the lectin pathway and regarding a potential unwanted modification in the amino-terminal glycosylation.

## C1INH in Human Myocardial I/R Injury

Although multiple experimental studies have demonstrated the beneficial effect of complement inhibition in general and of C1INH in particular, no complement inhibitor is currently approved for the treatment of AMI or other I/R injuries. Interventions specifically and exclusively targeting the lectin pathway of complement are lacking in humans, whereas the effect of C1INH has been already investigated in three clinical trials and one case series (Table 4). Similar to the above-mentioned experimental studies, only pdC1INH was used. Bauernschmitt et al. were the first to report on the experience of pdC1INH treatment in three patients undergoing emergency coronary artery bypass grafting (CABG) after failed percutaneous coronary intervention (74). All three patients developed severe postoperative myocardial dysfunction which impaired weaning from CPB. Termination of CPB in all three patients was associated with the administration of a single dose of pdC1INH, which led to improved left ventricular function and hemodynamic stabilization.

**Table 4:** Effect of C1INH in clinical trials of AMI.

Reference	Study type	Condition	Included patients	Treatment	Relevant findings
Bauernschmitt et al. (74)	Case series	Emergency CABG after myocardial infarction	3	pdC1-INH 2,000 IU before CPB weaning	Hemodynamic stabilization and improved ventricular function
de Zwaan et al. (75)	Open-label, dose-escalation study	STEMI	STEMI: 22 Historical controls: 18	pdC1-INH 50 or 100 IU/kg loading dose continuous infusion of 1.25–2 IU/kg/h for 48 h	↓ troponin T and creatine kinase levels compared with historical control cohort
Thielmann et al. (76)	Randomized, open-label study	STEMI followed by emergency CABG	57	pdC1-INH 40 IU/kg loading dose before aortic unclamping 20 IU/kg 6 h after surgery	↓ decline in C1INH activity and troponin T increase (only when treated less than 6 h after symptom onset)
Fattouch et al. (77)	Randomized, double-blind study	STEMI followed by emergency CABG	80	pdC1-INH 500 IU loading dose 10 min before aortic unclamping Continuous infusion of 500 IU over 3 h	↓ decline in C1INH levels, increase in C3a, C4a, and toponin I levels, length of stay in ICU and hospital, duration of mechanical ventilation ↑ cardiac contractile function

*CABG, coronary artery bypass grafting; CPB, cardiopulmonary bypass; ICU, intensive care unit; pdC1INH, plasma-derived C1 esterase inhibitor; STEMI, ST elevation myocardial infarction; AMI, acute myocardial infarction*.

In an open-label, dose-escalation study, de Zwaan et al. treated 22 patients with acute STEMI with pdC1INH during 48 h (75). The majority of patients had received antifibrinolytic therapy at least 1–2.5 h before C1INH administration, and only three patients underwent acute percutaneous coronary intervention. Drug-related adverse events were not observed, but creatine kinase and troponin T levels were reduced in comparison to a historical control population.

In a randomized, open-label study 28 patients undergoing emergency CABG after STEMI were treated with pdC1INH at aortic unclamping followed by another bolus dose 6 h later, and were compared with 29 similar patients receiving placebo and 10 patients undergoing elective CABG without evidence of recent STEMI (76). Again, drug-related adverse events were not observed. Administration of C1INH prevented the significant decline in C1INH activity (indicating consumption of C1INH) observed in placebo and control patients, whereas there was no difference in complement fragments. Interestingly, postoperative troponin T increase was only attenuated in patients receiving C1INH and surgical revascularization less than 6 h after symptom onset but not in patients with a longer ischemic interval.

In the largest study to date, Fattouch et al. randomized 80 patients with STEMI undergoing emergency CAGB to treatment with C1INH or placebo in a double-blind manner (77). C1INH was administered as an intravenous bolus before aortic unclamping followed by an intravenous infusion of 500 IU over 3 h after surgery. C1INH treatment resulted in significantly lower troponin T levels, an attenuated increase in complement activation fragments (C3a and C4a) and a shorter intensive care unit stay. Again, a significant decline of C1INH activity was only prevented in the active comparator group, and adverse events related to C1INH were not observed.

In summary, clinical studies of pdC1INH in human myocardial I/R suggest, that treatment of pdC1INH is safe and potentially effective in this setting. Of note, there is a lack of studies investigating C1INH in patients with AMI undergoing contemporary management with drug-eluting stents and state-of-the-art antithrombotic therapy. Mortality of AMI has significantly declined over the last decades as a result of modern drug and interventional treatment (78) and thus a potential benefit of pdC1INH as demonstrated in studies more than 10 years ago may not imply a similar positive effect in contemporary AMI patients. In addition, adverse drug reactions of C1INH administration must be meticulously evaluated in future studies given the potential of an increased bleeding but also thrombotic risk in the era of potent antithrombotic therapy and drug-eluting stents.

## Discussion

Acute myocardial infarction remains a leading cause of morbidity and mortality worldwide despite early and successful reperfusion strategies, which have been shown to significantly limit the size of the myocardial infarct and improve clinical outcomes. Although its existence remains controversial, reperfusion injury after restoration of blood flow has been regarded as a critical contributor to myocardial damage paradoxically limiting the beneficial effects of myocardial reperfusion. With respect to the modulation of the complement response, several complement inhibitors targeting different proteins of the complement cascade have been successfully investigated in experimental models of myocardial I/R injury (79). These models have underscored the potential of attenuating myocardial tissue damage and ventricular remodeling by complement inhibition. However, with the exception of a single randomized placebo-controlled phase 3 trial, there is a lack of high-quality clinical studies of complement inhibition in human AMI (8). In addition, single-target interventions such as inhibition of C5 are probably inadequate to address the manifold inflammatory reactions *via* several cascades and pathways after AMI. For example, pexelizumab only attenuated the increase in C5 and interleukin-6 levels but had no impact on the increase and decrease of several other pro- and anti-inflammatory proteins, respectively (80).

## Future Perspectives

Based on the data as outlined above, we would like to suggest two potential strategies of complement inhibition for future clinical trials that are not mutually exclusive.

The first strategy involves targeted inhibition of the lectin pathway of complement. As the activity of the lectin pathway essentially depends on MASP-2 as the central enzyme (81), selective inhibition of MASP-2 shortly after myocardial ischemia and before reperfusion seems like an obvious next step. However, several caveats have to be considered apart from the availability of a suitable anti-MASP-2 antibody for future clinical trials.

MASP-2 inhibition, although effective in the above mentioned animal model, may be partially bypassed by the function of at least two lectin pathway proteins, such as MBL and MASP-1. For example, targeted MAPS-2 inhibition will not impact on several pro-inflammatory functions of MASP-1 such as endothelial cell activation (82) and the promotion of clot formation (54) *via* cleavage of thrombin substrates and the activation of platelets (68). Most importantly, evidence regarding the significance of the lectin pathway in human AMI is still premature and limited. Hence, a strategy that again only targets a single protein of the complement system (although further upstream in the complement cascade as in the pexelizumab trials) may be of limited or uncertain benefit in clinical trials of AMI. The complement system has also been implicated as a mediator of regenerative processes after myocardial tissue injury (83), and hence it is imperative to study the effect of short-term complement inhibition on outcomes after at least 30 days.

Treatment with C1INH may be regarded as a potential solution to this challenge. There are several advantages of using C1INH compared with isolated MASP-2 inhibition but also some caveats. In contrast to the previously mentioned strategies of complement inhibition, C1INH is a multiple-target, multiple-action inhibitor. Myocardial I/R injury is not mediated or caused by a single protein or even pathway, rather the opposite is true, i.e., several pathways are activated simultaneously and act in parallel. In addition, the relative contribution of each involved protein and pathway to the net damage is unknown and may vary in the line with the significant heterogeneity of AMI itself and of the diverse patient populations that suffer from AMI.

Evidence from multiple experimental studies mentioned in the present review point to a relevant and protective effect of C1INH in myocardial I/R injury. Moreover, results from small clinical trials, though conducted more than 10 years ago, seem to confirm findings from animal models. Similarly important as effectiveness is the fact that adverse drug reactions of C1INH administration were not reported in the setting of human AMI (75–77) and of acute rejection following renal transplantation (84–86).

Potential disadvantages of C1INH include unwanted effects when interfering with the coagulation and the fibrinolysis system at the same time. As a matter of fact, thromboembolic complications were noted in neonates receiving high-dose C1INH during cardiopulmonary bypass surgery and in an animal model of myocardial I/R injury (33). However, there was no safety signal in the above-mentioned clinical trials of C1INH.

When designing clinical trials of C1INH in AMI several aspects and pitfalls have to be addressed such as the required duration of treatment. In the previous pexelizumab trial in STEMI patients, complement inhibition was sustained for at least 24 h with its activity having returned to baseline after 48 h. However, elevated serum complement levels have been demonstrated during the first 10 days after AMI (87).

The second question is which type of C1INH should be investigated in future clinical trials of AMI. PdC1INH seems to be the obvious choice since it has been utilized in every experimental and human myocardial I/R injury study to date. Another advantage is its significantly longer half-life compared with Conestat alfa (30 vs. 3 h) (88). However, Conestat alfa significantly decreased ischemic damage when administered up to 18 h after induction of cerebral ischemia and reperfusion, whereas pd1INH was only effective when given at the time of reperfusion (31, 89). Similarly, the formation of plasmatic functional MBL/MASP-2 complexes after transient cerebral ischemia was attenuated only in mice receiving Conestat alfa but not pdC1INH.

Another important aspect involves the requirement of inhibiting target proteases and non-protease targets at the site of acute inflammation. To maximize effectiveness and minimize adverse reactions, an ideal C1INH preparation should exert its inhibitory function preferably or exclusively in ischemic and/or reperfused myocardial tissue similar to the concept of targeting cancer cells by mAbs. In contrast to cancer, the speed of inactivation is also crucial in I/R injury, as for example MBL-MASP-1/-2 complexes clustered together on ischemic endothelial or myocardial cells may escape inactivation by C1INH long enough to activate downstream effector molecules.

Whereas the protein structure is identical, the glycosylation pattern of Conestat alfa at the amino-terminal domain is markedly different from pdC1INH preparations (32). In particular, exposed mannose residues are significantly more prevalent in Conestat alfa compared with pdC1INH (15 vs. <1%), which may influence the binding preference toward lectins and in particular to MBL. Indeed, Gesuete et al. demonstrated high-affinity binding of Conestat alfa to MBL but not of pdC1INH (31). Although this may not directly influence the degree of inhibition by its serpin domain, it may potentially impact on the speed and location of inhibition in the human body. By binding to MBL, Conestat alfa may be hijacked to the primary site of inflammation, where it may immediately inhibit its target proteases before they are able to activate downstream effector molecules. By contrast, pdC1INH is certainly able to limit random complement activation in plasma but may allow considerable escape of inactivation of complement complexes at the tissue level. Interestingly, Conestat alfa remained confined on the ischemic endothelial wall in co-localization to MBL, whereas pdC1INH was also found in the area around the ischemic vessels (31).

However, due to the lack of comparative studies of pdC1INH vs. Conestat alfa in myocardial I/R injury it is premature to draw any definitive conclusions about any difference in efficacy of the two preparations in the setting of AMI.

## Summary and Conclusion

In this review, we have summarized current concepts and evidence addressing the role of the lectin pathway as a potent regulator of myocardial I/R injury in murine models and the human setting. As it still remains to be determined if administration of a complement inhibitor after myocardial ischemia and before reperfusion is effective, we may have to leave the beaten path and “should strike out on new paths” (John D. Rockefeller, 1839–1937) avoiding single-target interventions and investigate pleiotropic compounds such as the natural inhibitor of the lectin pathway, C1INH, that also interferes with other important pro-inflammatory pathways. Given the evidence from several animal models and previous small clinical trials and the lack of major concerns regarding adverse events, there is ample reason to embark on larger clinical trials with C1INH in STEMI patients. In our opinion, ameliorating myocardial I/R injury by targeting the lectin pathway of complement remains a valid option for future therapeutic interventions.

## Author Contributions

All authors listed have made a substantial, direct, and intellectual contribution to the work including drafting and critical revising the article and approved it for publication.

## Conflict of Interest Statement

MO is the principal investigators and MT is a co-investigator of an investigator initiated clinical trial (NCT02869347; https://clinicaltrials.gov/ct2/show/NCT02869347) of Conestat alfa in the prevention of contrast-induced renal damage and have received an investigator initiated research grant from Pharming Technologies B.V., the manufacturer of Conestat alfa, for this trial. Pharming Technologies B.V. had no influence on the design and content of this review. The views expressed here are the responsibility of the authors only. AP declares no conflict of interest.
